# Strain Analysis from Transverse CMR Cine Imaging in Congenital Heart Disease: Feasibility, Reproducibility, and Comparison to Global Longitudinal Strain †

**DOI:** 10.3390/healthcare14030411

**Published:** 2026-02-06

**Authors:** Victoria Zieschang, Peter Kramer, Collin Götze, Sebastian Kelle, Regina Stegherr, Alireza Khasheei, Felix Berger, Johannes Nordmeyer, Titus Kühne, Sarah Nordmeyer, Marie Schafstedde

**Affiliations:** 1 Department of Radiology, Charité—Universitätsmedizin Berlin, Augustenburger Platz 1, 13353 Berlin, Germany; victoria.zieschang@charite.de; 2 Department of Congenital Heart Disease—Pediatric Cardiology, Deutsches Herzzentrum der Charité—Medical Heart Center of Charité, Augustenburger Platz 1, 13353 Berlin, Germany; 3 Department of Cardiology, Angiology and Intensive Care Medicine, Deutsches Herzzentrum der Charité—Medical Heart Center of Charité, Augustenburger Platz 1, 13353 Berlin, Germany; 4 German Centre for Cardiovascular Research, Deutsches Zentrum für Herz-Kreislauf-Forschung, Partner Site Berlin, 10785 Berlin, Germany; 5 Institute of Biometry and Clinical Epidemiology, Charité—Universitätsmedizin Berlin, Charitéplatz 1, 10117 Berlin, Germany; 6 Institute for Cardiovascular Computer-Assisted Medicine, Deutsches Herzzentrum der Charité—Medical Heart Center of Charité, Augustenburger Platz 1, 13353 Berlin, Germanysarah.nordmeyer@med.uni-tuebingen.de (S.N.); 7 Department of Pediatric Cardiology, Pulmonology and Pediatric Intensive Care Medicine, University Children’s Hospital Tübingen, Hoppe-Seyler-Str. 1, 72076 Tübingen, Germany; 8 Department of Diagnostic and Interventional Radiology, University Hospital Tübingen, 72076 Tübingen, Germany; 9 Berlin Institute of Health, Charité—Universitätsmedizin Berlin, Charitéplatz 1, 10117 Berlin, Germany

**Keywords:** CMR, congenital heart disease, global longitudinal strain, Fontan, tetralogy of Fallot, transverse oriented cine imaging, reproducibility

## Abstract

**Background**: Global longitudinal strain (GLS), derived from long-axis cine images (LAX), is a sensitive marker for myocardial dysfunction and a strong predictor for clinical events and future ventricular deterioration. In patients with complex congenital heart disease (CHD) transverse-oriented cine imaging is part of the standard cardiac magnetic resonance (CMR) protocol. We aimed to study the feasibility and reproducibility of strain measurements derived from transverse-oriented cine images (Transverse Strain (TrS)) and compare them with standard GLS. **Methods**: We retrospectively analyzed CMR cine images from 40 patients (n = 20 Fontan, n = 20 Tetralogy of Fallot (ToF)) and 10 healthy controls. Strain analysis was performed in every subject using both the conventional GLS and the TrS approach. **Results**: TrS showed high intra- and interobserver reproducibility in patients with CHD (intraclass correlation coefficient (ICC) > 0.75, *p* < 0.05). Intermethod agreement between TrS and GLS was strong in Fontan patients and in the right ventricle (RV) of ToF patients (ICC > 0.75) but showed a positive bias for TrS in the left ventricle (LV) of ToF patients (mean difference = 5.03) and in both ventricles of healthy controls (mean difference LV = 5.36, RV = 4.01). **Conclusions**: TrS is feasible and reproducible and may offer a new methodological approach for strain assessment, especially in CHD patients with univentricular physiology and ToF patients. Further studies are needed to validate this new approach and perform correlations to clinical outcomes.

## 1. Introduction

Global longitudinal strain (GLS) derived from feature-tracking in cardiac magnetic resonance (CMR) imaging is a sensitive marker for subclinical myocardial dysfunction and an established predictor of adverse outcomes across various cardiac diseases [1,2,3,4]. In line with this, the large prospective HERZCHECK trial further highlighted the clinical relevance of CMR-derived GLS by using it as the central imaging parameter for screening and risk stratification of asymptomatic individuals at risk for heart failure [5]. Standardized GLS quantification is typically performed from left ventricular (LV) long-axis (LAX) cine views (2-, 3-, and 4-chamber), while right ventricular (RV) strain is conventionally derived from the 4-chamber view [6,7,8].

In CMR protocols for patients with complex congenital heart disease (CHD), such as those with univentricular physiology, standardized LAX cine acquisitions for GLS can be technically challenging or suboptimal to perform due to the presence of a morphologic right systemic ventricle, abnormal ventricular orientation, atypical looping, and non-standard atrioventricular alignment [9,10,11]. As a result, consistent and reproducible LAX planes are often difficult to obtain, limiting the applicability of conventional GLS analysis. Although consensus recommendations outline disease-specific CMR protocols in CHD, including long-axis views for tetralogy of Fallot (ToF) and Fontan patients [9] protocols vary between centers and are frequently adapted to local expertise in CMR techniques, patients’ anatomy, surgical history, and clinical indication [12], which can lead to incomplete or absent LAX datasets. For example, in patients with ToF, imaging typically focuses on right-ventricular size, function, and the right-ventricular outflow tract [13], so dedicated LAX acquisitions are often not included in standard protocols.

These anatomical and protocol-related limitations are also reflected in the literature, where the absence of suitable long-axis datasets has led Strodka et al. [14] to rely on transverse cine stacks for strain estimation. The group used axial cine images as substitutes for missing four-chamber views in Fontan patients, highlighting the broader clinical need for an alternative, anatomy-independent orientation for myocardial strain assessment in CHD.

Transverse-oriented cine images, however, are routinely acquired as part of standard CMR protocols across all CHD subtypes [9]. This imaging plane provides full ventricular coverage independent of cardiac orientation and allows for comprehensive assessment of both ventricles and adjacent vascular structures within a single stack [10]. In patients with ToF, transverse-oriented cine images capture the entire right ventricle and outflow tract, which are critical for postoperative evaluation, whereas in patients with Fontan circulation, they enable visualization of the complex systemic and pulmonary venous pathways and extracardiac conduits within one continuous anatomical dataset [11,15,16].

Since transverse-oriented cine images have been—and continue to be—routinely acquired in CMR examinations of patients with CHD, whereas LAX images have not necessarily been, it is of interest to determine whether strain derived from transverse-oriented cine images (Transverse Strain (TrS)) may also serve as a feasible and reliable marker for myocardial contractility.

Accordingly, the aim of this study was to evaluate left- and right-ventricular strain derived from transverse-oriented cine images and, for the first time, to assess its reproducibility and compare it with conventional GLS in patients with CHD (including univentricular anatomy and ToF) as well as in healthy controls.

## 2. Materials and Methods

### 2.1. Study Population and Design

This retrospective study included 50 subjects who underwent CMR at our institution between 2015 and 2021, with CMR protocols that included LAX cine images and cine images in transverse slice orientation suitable for strain analysis. The study population consisted of three groups: 20 patients after Fontan operation (12 female, 8 male), 20 patients after ToF repair (11 female, 9 male), and 10 healthy controls (6 female, 4 male) without known cardiovascular disease. Healthy controls were selected from individuals referred for CMR to exclude structural heart disease and who showed normal cardiac anatomy and function.

All patients or their legal guardians gave informed consent by signing the institution’s broad consent form for retrospective analysis of clinical and imaging data.

Inclusion criteria were the availability of transverse-oriented cine images and LAX (2-, 3-, and 4-chamber views) cine acquisitions of sufficient quality for feature-tracking strain analysis. Exclusion criteria included incomplete cine datasets, poor image quality (e.g., severe artifacts such as motion artifacts or device-related artifacts in patients with pacemakers or implantable cardioverter-defibrillators), or CHD diagnoses that did not fit the predefined groups. Mean age was 16 ± 6.5 years in the Fontan group, 26 ± 13.4 years in the ToF group, and 15 ± 5.1 years in the control group. In the Fontan group, 75% of patients had a systemic left ventricle, 25% a systemic right ventricle. An overview of the patient characteristics, including sex, age, volumetric parameters, ejection fraction, and cardiac index, is provided in Table 1. Additional detailed clinical characteristics of the Fontan cohort are provided in the Supplementary Material (Table S1).

### 2.2. CMR Acquisition and Functional Analysis

All included routine clinical CMR examinations were performed on a 1.5-T scanner (Achieva, Philips Healthcare, Best, The Netherlands). The datasets typically comprised cine steady-state free-precession sequences acquired in short-axis (SAX) orientation for ventricular volumetry, LAX views (2-, 3-, and 4-chamber), and transverse cine image stacks. Typical acquisition parameters showed only minor variations between examinations and included a repetition time (TR) of approximately 3.0 ms, an echo time (TE) of approximately 1.5 ms, an in-plane spatial resolution of about 1.0–1.5 × 1.0–1.5 mm^2^, a slice thickness of 6–8 mm, and approximately 20–30 cardiac phases per cardiac cycle.

Functional analysis was performed using QMass (v1.4.0.158, Medis Medical Imaging Leiden, The Netherlands). End-systolic (ES) and end-diastolic (ED) phases were visually selected on SAX and transverse-oriented cine images. Endocardial and epicardial contours were manually traced from base to apex for volumetric analysis of both ventricles in ToF patients and healthy controls, as well as of the systemic ventricle and—if distinguishable—the hypoplastic ventricle in Fontan patients. Papillary muscles and trabeculae were excluded from ventricular volumes and from ventricular mass calculations. Ventricular end-diastolic volume, end-systolic volume, and mass were derived from SAX stacks as the standard reference for functional assessment. Stroke volume and ejection fraction of the ventricles were calculated automatically. Height and weight were obtained to determine body surface area (BSA), and ventricular volumes and mass were indexed to BSA. Cardiac index was calculated accordingly.

### 2.3. Strain Analysis/CMR Feature-Tracking

CMR feature-tracking analysis was performed using QStrain (QSTRAIN 1.4.0.158, Medis Medical Imaging Leiden, The Netherlands).

CMR feature-tracking was performed using two methods in every subject:

1. GLS, expressed as a negative value, represents base-apex shortening and is derived by averaging the peak strain values of individual segments using the 17-segment model [17]. GLS analysis of the systemic or left ventricle was performed using LAX (2-, 3-, and 4-chamber) cine images, and GLS of the hypoplastic or right ventricle from a single LAX (4-chamber) image. Analysis was conducted automatically and manually by tracing the endocardial and epicardial borders in ES and ED across all required planes.
2. TrS was performed using transverse-oriented cine images. For standardizing purposes, we chose to include only images with clearly detectable atria, ventricles, and atrioventricular plane in both ES and ED. The atrioventricular plane served as the anatomical base for contouring. Slices in which the atrioventricular plane was not clearly identifiable in either phase were excluded.

From the available transverse-oriented cine images, up to three slices meeting all criteria were selected independently by two observers. A maximum of three slices was predefined to ensure methodological feasibility and reasonable post-processing time. Depending on individual anatomy and image quality, one to three eligible slices were available. The mean number (±SD) of analyzed slices was 3.0 ± 0 for Fontan patients (median 3), 2.7 ± 0.5 for ToF patients (median 3), and 2.2 ± 0.4 for controls (median 2) for observer 1. Corresponding values for observer 2 were 3.0 ± 0 for Fontan patients (median 3), 2.9 ± 0.3 for ToF patients (median 3), and 2.4 ± 0.8 for controls (median 3).

Endocardial and epicardial borders were manually traced from the atrioventricular plane (base) to the apex in ES and ED using QStrain. In patients with septal defects, an imaginary contour line was drawn across the defect if needed. TrS was calculated as the mean of all strain values from the selected slices.

**Figure 1:**
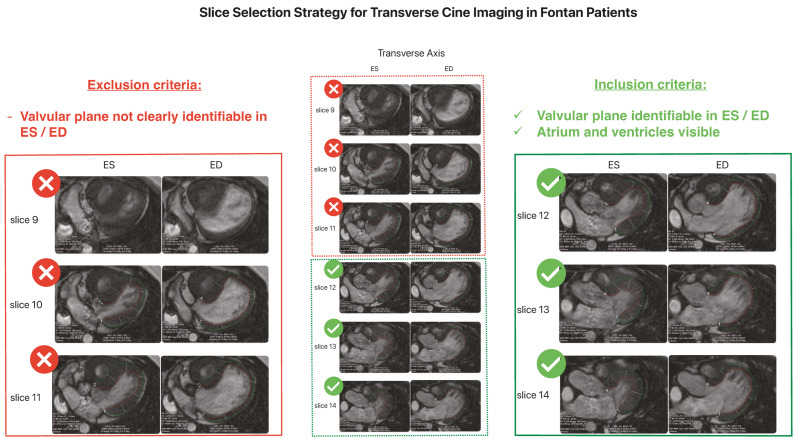
Slice selection strategy for transverse strain (TrS) analysis using transverse-oriented cine images. Example slices from transverse cine images in end-systole (ES) and end-diastole (ED). Slices were excluded if the atrioventricular plane was not clearly identifiable in either phase (slices 9–11). Inclusion required a clear visualization of atria, ventricles, and the atrioventricular plane in both ES and ED (slices 12–13). From eligible slices, up to three were selected for strain analysis.

This process is illustrated in Figure 1, which shows the slice selection strategy from transverse-oriented cine images, including typical inclusion and exclusion criteria.

### 2.4. Statistical Analysis

All analyses were performed using IBM SPSS Statistics, V29.0.2.0. A *p*-value < 0.05 was considered statistically significant.

**Figure 2:**
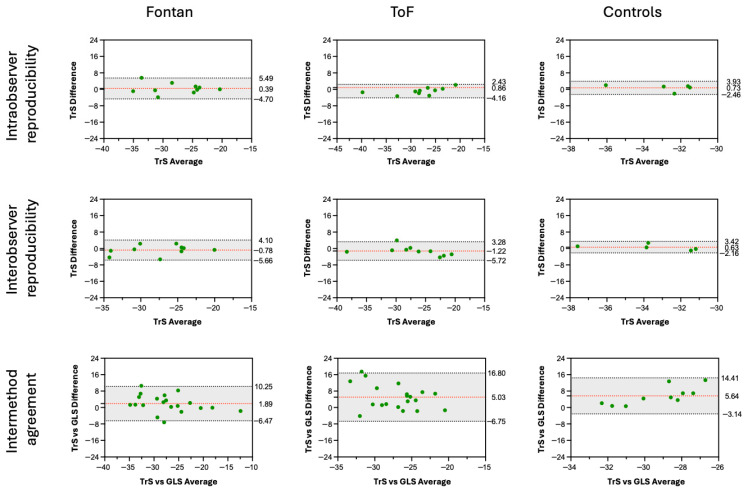
Bland–Altman plots of endocardial transverse strain (TrS) for the left ventricle (in ToF patients and controls) or the systemic ventricle (in Fontan patients). The plots illustrate intraobserver reproducibility (top row), interobserver reproducibility (middle row), and intermethod agreement (bottom row) across the three study groups: Fontan (left column), ToF (middle column), and healthy controls (right column). Each plot displays the mean difference (red dashed line) and 95% limits of agreement (black dotted lines), green dots represent individual measurements. GLS = global longitudinal strain; ToF = tetralogy of Fallot; TrS = transverse strain.

The agreement between strain measurements derived from LAX and transverse cine images, as well as intra- and interobserver reproducibility of TrS analysis, is summarized descriptively for each patient group based on the intraclass correlation coefficient (ICC), mean difference, limits of agreement, and Pearson correlation coefficient (r), visualized using Bland–Altman plots (Figure 2).

The following levels of agreement were used: excellent for ICC > 0.75, good for ICC 0.6–0.74, fair for ICC 0.4–0.59, poor for ICC < 0.4 [18].

Half of each patient group (10 Fontan, 10, ToF, 5 healthy controls) was reanalyzed 6 months after the first analysis by the first observer, blinded to the results of the first analysis. The same dataset (10 Fontan, 10, ToF, 5 healthy controls) was analyzed by a second observer who was blinded to the results of the first observer.

Detailed results are summarized in Section 3. Additional TrS analyses are available in the Supplementary Materials (Tables S2–S4), showing similar trends.

## 3. Results

### 3.1. Intra- and Interobserver Reproducibility

Intra- and interobserver reproducibility of TrS analysis was good to excellent across all patient groups, with consistently high reproducibility in Fontan and ToF patients (Table 2 and Table 3). In Fontan patients, reproducibility was excellent for both single ventricle (SV) and hypoplastic ventricle (HV) (all ICC ≥ 0.82). Similarly, ToF patients showed excellent agreement for the LV (intra-/interobserver ICC = 0.95/0.91) and good to excellent agreement for the RV (ICC = 0.81/0.80). In the control group, intraobserver reproducibility was excellent for the RV (ICC = 0.96) and good for the LV (ICC = 0.68, *p* = 0.08). Interobserver agreement in controls was lower overall, with excellent reproducibility in the LV (ICC = 0.86).

### 3.2. Intermethod Comparison Between TrS and GLS

Intermethod agreement between TrS and GLS varied across patient groups (Table 4). Mean differences were calculated as GLS − TrS; therefore, positive values indicate more negative TrS values compared with GLS. In Fontan patients, good to excellent agreement between both methods was observed for both ventricles, especially in the systemic ventricle (ICC = 0.75), with only small systematic differences. In ToF patients, agreement between TrS and GLS was excellent for the RV (ICC = 0.78), whereas LV agreement was poor, with TrS values showing a notably more negative strain than GLS (mean difference = 5.03). In healthy controls, agreement between both methods was poor, with weak correlations and consistently more negative TrS values compared to GLS. Bland–Altman plots illustrating these findings are shown in Figure 2, with additional analyses provided in the Supplementary Materials (Figures S1 and S2).

## 4. Discussion

This study demonstrates that strain measurements derived from transverse cardiac cine images (TrS) offer high intra- and interobserver reproducibility and might be an applicable method for quantifying myocardial contractility and deformation. In Fontan patients with either a morphological left or right systemic ventricle, and in the right ventricle of Fallot patients, TrS values were even comparable to intraindividual GLS values. In the left ventricle of ToF patients and in both ventricles of healthy controls, however, TrS values were significantly more negative than GLS values.

### 4.1. Reproducibility

TrS measurements demonstrated good to excellent intra- and interobserver reproducibility across all patient groups, reinforcing their reliability for possible clinical evaluation and use. The standardized use of up to three well-defined transverse cine images per subject contributed to this consistency and may serve as a guideline for future application.

### 4.2. Intermethod Agreement and Systematic Bias

Good agreement was seen between TrS and GLS values in the ventricles of patients with Fontan circulation and in the RV of patients with repaired ToF. This consistent intermethod agreement, although unexpected at first, may be explained by an altered myocyte arrangement in these ventricles resulting from congenital abnormalities and remodeling induced by volume and/or pressure load. In Fallot patients after surgical correction, altered ventricular geometry, RV remodeling due to chronic pressure and/or volume overload, and diffuse, partly asynchronous deformation patterns have been described [19,20,21,22]. Similarly, in Fontan patients, non-uniform and directionally inconsistent myocardial motion of the single ventricle has been reported [23,24]. These abnormalities in myocyte arrangement might lead to a less organized contraction pattern compared with structurally normal ventricles and may explain why strain measurements, although derived from different image orientations (standard LAX cine images vs. transverse-oriented cine images), yield similar values.

Together, these findings suggest that in CHD patients with fundamentally altered myocardial mechanics, strain measurements become less dependent on imaging orientation. This may account for the high intermethod agreement observed in both the RV of ToF patients and in the univentricular hearts of Fontan patients. This interpretation is based on the present findings and should be interpreted as a hypothesis, given that it was not specifically evaluated within the current study design.

In contrast, systematic differences with more negative TrS than GLS and poor correlation were observed in the left ventricle of ToF patients and in both ventricles of healthy controls. This indicates that TrS may not be suitable for direct comparison with GLS in structurally normal ventricles and therefore should be regarded as an independent deformation parameter rather than a substitute for GLS. Importantly, agreement estimates in the control group should be interpreted with caution, as the small sample size and the inherently low interindividual variability in healthy subjects may affect ICC calculations and may partly explain the negative ICC values observed.

The consistently more negative strain values observed with TrS likely reflect a combination of factors, including inclusion of myocardial regions with high contractility (e.g., the lateral wall) [25], orientation misalignment of strain vectors relative to fiber direction, and the broader deformation pattern sampled in transverse planes.

Standard longitudinal cine images primarily capture longitudinal deformation, whereas transverse-oriented cine images intersect the ventricular myocardium orthogonally and may capture components of longitudinal, circumferential, and rotational motion. This broader deformation coverage, together with the inclusion of myocardial regions with high contractility, such as the lateral wall, may explain the differences seen between TrS and GLS values [25].

### 4.3. Clinical Relevance

GLS has demonstrated prognostic significance in various cardiovascular diseases and is known to outperform traditional volumetric parameters like ejection fraction in detecting early myocardial dysfunction [1,2]. Although consensus recommendations provide guidance on disease- and anatomy-specific CMR protocols in congenital heart disease [9], including the recommended long-axis views for ToF or Fontan patients—these guidelines remain recommendations rather than strict standards. In clinical practice, CMR protocols are frequently adapted to the individual patient’s anatomy, surgical history, and clinical questions, and minimized in duration, which can result in incomplete or absent long-axis datasets even when they are formally recommended. Accordingly, Strodka et al. [14] used transverse cine images when long-axis datasets were not available. Such approaches demonstrate that missing LAX images are a practical reality in some CHD cohorts and underline the need for validated alternative strain methods.

By enabling strain measurements in patients for whom conventional methods are limited or unavailable, TrS measurements could enhance risk stratification, monitoring, and treatment planning in CHD and may provide an additional marker of myocardial function, particularly for the early detection of ventricular dysfunction prior to changes in ejection fraction.

Another noteworthy advantage of using TrS analysis instead of or next to GLS in CHD would be the possibility to perform large-scale retrospective longitudinal studies, since transverse cardiac cine images are largely available in multiple sites around the world for multiple years, offering a potential large cohort to study myocardial contractility and clinical outcome [9,26,27].

Thus, TrS provides a novel and practical solution to extend strain-based evaluation to patients who would otherwise be excluded from strain analysis. Further longitudinal studies are required to assess whether TrS values correlate with clinical outcomes and adverse events in this specific population.

### 4.4. Limitations

This study has several limitations. The relatively small sample size, particularly in the control group, may have limited statistical power and increased the risk of type II error. Moreover, the retrospective single-center design may have introduced selection bias and limited the generalizability of the results.

Methodological limitations are mainly related to the TrS approach. In contrast to the standard 17-segment GLS model, TrS is based on a limited number of transverse slices and does not provide complete ventricular coverage, which may have influenced strain measurements. Although slice selection followed predefined criteria, a certain degree of observer dependence cannot be fully excluded. In addition, strain analysis is software-dependent and therefore not interchangeable across different post-processing platforms.

Finally, all strain values were derived using a feature-tracking approach. Consequently, the present results reflect feature-tracking–based strain and are not directly comparable to strain measurements obtained with other techniques, such as myocardial tagging. Furthermore, the lack of established reference values for TrS currently limits its immediate clinical applicability.

### 4.5. Validation and Future Directions

While the results are promising, broader validation remains essential. Larger studies across different institutions and patient populations are needed to confirm the method’s reliability and to establish population-specific reference values for TrS. The study also emphasizes the need for tailored software tools, such as specific segmentation models and algorithms optimized for TrS analysis, to improve applicability.

While several studies have established the feasibility and prognostic value of GLS in pediatric and adult CHD [3,23,28,29], to date, no study has evaluated the use of TrS for strain assessment in this population. The present study adds novel evidence, demonstrating that TrS is not only reproducible but also correlates well with the gold standard in selected CHD subgroups. Reported ICC values for reproducibility compare favorably with published LAX-based studies, further supporting the methodological validity of TrS measurements [30,31].

## 5. Conclusions

This study demonstrates that TrS is a feasible and reproducible method for myocardial strain analysis in patients with CHD, particularly in those with non-standard anatomy such as Fontan circulation or the right ventricles of patients with corrected ToF. Across all study groups, TrS showed high intra- and interobserver reproducibility, confirming the consistency of the method, thereby underscoring its utility for longitudinal monitoring and its suitability for standardized application in structurally complex anatomies.

Intermethod agreement between TrS and GLS was high in Fontan patients and in the RV of Fallot patients, supporting the use of TrS as a potential alternative to GLS in these subgroups. In contrast, TrS yielded consistently more negative strain values in both ventricles of healthy controls and in the LV of Fallot patients, indicating that in these groups, TrS should not be used interchangeably with GLS and should be instead interpreted as an independent deformation parameter.

To further establish the clinical role of TrS, future studies should define reference values across diverse populations and cardiac pathologies, explore the relationship between TrS and clinical outcomes, and validate the method in larger, multicenter cohorts.

## Figures and Tables

**Table 1:** Patient demographics and functional cardiac magnetic resonance-based parameters.

Patient Characteristics	Fontan n = 20	ToF n = 20	Controls n = 10
Demographics			
Sex, female n (%)	12 (60)	11 (55)	6 (60)
Age at MRI (years), mean ± SD	16 ± 6.5	26 ± 13.4	15 ± 5.1
Height (cm), mean ± SD	150.1 ± 18.6	159.6 ± 21.0	156.2 ± 17.4
Weight (kg), mean ± SD	47.1 ± 19.3	63.1 ± 25.8	51.8 ± 20.8
BSA, mean ± SD	1.4 ± 0.3	1.7 ± 0.4	1.5 ± 0.4
Heart rate (bpm), mean ± SD	74.3 ± 23.2	79.1 ± 9.1	79.6 ± 13.1
Functional parameters			
EDVi (mL/m^2^), mean ± SD	82.2 ± 26.9	78.6 ± 14.9	81.2 ± 6.6
ESVi (mL/m^2^), mean ± SD	42.5 ± 24.4	33.3 ± 7.2	34.1 ± 5.9
SVi (mL/m^2^), mean ± SD	39.4 ± 13.0	45.3 ± 8.7	47.1 ± 4.5
EF (%)	49.8	57.8	58.2
CI (L/min/m^2^), mean ± SD	3.0 ± 1.0	3.4 ± 0.6	3.7 ± 0.4

Data are presented as numbers (percentage) or mean ± standard deviation. Volumetric and functional parameters refer to the systemic ventricle in Fontan patients and the left ventricle in ToF and controls. BSA = body surface area, CI = cardiac index, EDVi = end-diastolic volume indexed to BSA, EF = ejection fraction, ESVi = end-systolic volume indexed to BSA, SVi = stroke volume indexed to BSA, ToF = tetralogy of Fallot.

**Table 2:** Intraobserver reproducibility of TrS analysis in Fontan, ToF, and control subjects.

Group	1st Meas. TrS	2nd Meas. TrS	Mean Difference	Limits of Agreement	ICC (95% CI)	r	*p*-Value
**Fontan**							
SV	−27.5 ± 4.9	−27.9 ± 5.2	0.39	−4.70–5.49	0.87 (0.55–0.97)	0.87	0.001
HV	−22.5 ± 10.8	−21.9 ± 7.6	−0.53	−11.47–10.41	0.82 (0.34–0.96)	0.88	0.004
**ToF**							
LV	−28.5 ± 5.8	−27.6 ± 4.8	−0.86	−4.16–2.43	0.95 (0.81–0.99)	0.97	<0.001
RV	−22.3 ± 4.1	−21.9 ± 4.3	−0.42	−5.44–4.60	0.81 (0.41–0.95)	0.81	0.004
**Controls**							
LV	−32.5 ± 1.7	−33.3 ± 2.3	0.73	−2.46–3.93	0.68 (−0.30–0.96)	0.70	0.19
RV	−30.3 ± 4.5	−29.3 ± 4.1	−1.02	−3.34–1.30	0.96 (0.69–1.00)	0.97	0.007

The table presents transverse strain (TrS) values, reported as mean ± SD, measured twice by the same observer, including mean difference (bias), limits of agreement, ICC (with 95% CI), Pearson’s correlation coefficient, and *p*-values. HV = hypoplastic ventricle; ICC = intraclass correlation coefficient; LV = left ventricle; meas. = measurement; r = Pearson’s correlation coefficient; RV = right ventricle; SD = standard deviation; SV = systemic ventricle; ToF = tetralogy of Fallot; TrS = transverse strain.

**Table 3:** Interobserver reproducibility of TrS analysis in Fontan, ToF, and control subjects.

Group	1st Obs. TrS	2nd Obs. TrS	Mean Difference	Limits of Agreement	ICC (95% CI)	r	*p*-Value
**Fontan**							
SV	−27.9 ± 5.2	−27.1 ± 4.5	−0.78	−5.66–4.10	0.87 (0.56–0.97)	0.88	<0.001
HV	−21.9 ± 7.6	−21.1 ± 7.7	−0.87	−5.25–3.51	0.96 (0.80–0.99)	0.96	<0.001
**ToF**							
LV	−27.6 ± 4.8	−26.4 ± 5.9	−1.22	−5.72–3.28	0.91 (0.68–0.98)	0.93	<0.001
RV	−21.9 ± 4.3	−19.8 ± 3.7	−2.15	−7.26–2.97	0.80 (0.39–0.95)	0.80	0.005
**Controls**							
LV	−33.3 ± 2.3	−33.9 ± 3.0	0.63	−2.17–3.42	0.86 (0.15–0.98)	0.89	0.04
RV	−29.3 ± 4.1	−26.2 ± 3.4	−3.03	−9.92–3.86	0.57 (−0.45–0.94)	0.58	0.31

The table shows transverse strain (TrS) measurements, reported as mean ± SD, obtained independently by two observers, including mean difference (bias), limits of agreement, ICC (95% CI), Pearson’s correlation coefficient, and *p*-values. HV = hypoplastic ventricle; ICC = intraclass correlation coefficient; LV = left ventricle; obs. = observer; r = Pearson’s correlation coefficient; RV = right ventricle; SD = standard deviation; SV = systemic ventricle; ToF = tetralogy of Fallot; TrS = transverse strain.

**Table 4:** Intermethod agreement between GLS and TrS in Fontan, ToF, and control subjects.

Group	GLS (Mean ± SD)	TrS (Mean ± SD)	Mean Difference	Limits of Agreement	ICC (95% CI)	r	*p*-Value
**Fontan**							
SV	−26.3 ± 5.4	−28.2 ± 6.7	1.89	−6.47–10.25	0.75 (0.48–0.89)	0.77	<0.001
HV	−20.2 ± 11.6	−20.4 ± 9.5	0.18	−16.48–16.84	0.68 (0.26–0.89)	0.69	0.006
**ToF**							
LV	−24.6 ± 3.7	−29.6 ± 5.4	5.03	−6.75–16.8	0.16 (−0.30–0.55)	0.17	0.48
RV	−25.2 ± 4.2	−23.7 ± 3.7	−1.54	−6.69–3.62	0.78 (0.53–0.91)	0.79	<0.001
**Controls**							
LV	−26.5 ± 3.9	−31.8 ± 1.7	5.36	−3.76–14.47	−0.20 (−0.72–0.46)	−0.27	0.45
RV	−27.6 ± 5.1	−31.6 ± 3.5	4.01	−4.79–12.81	−0.48 (−0.18–0.84)	−0.51	0.14

The table presents intermethod agreement between GLS and transverse strain (TrS), reported as mean ± SD for each method, along with mean difference (bias), limits of agreement, intraclass correlation coefficient (ICC; 95% CI), Pearson’s correlation coefficient, and *p*-values. GLS = global longitudinal strain; HV = hypoplastic ventricle; ICC = intraclass correlation coefficient; LV = left ventricle; r = Pearson’s correlation coefficient; RV = right ventricle; SD = standard deviation; SV = systemic ventricle; ToF = tetralogy of Fallot; TrS = transverse strain.

## Data Availability

The original contributions presented in this study are included in the article/Supplementary Materials. Further inquiries can be directed to the corresponding author.

## Supplementary Materials

The following supporting information can be downloaded at: https://www.mdpi.com/article/10.3390/healthcare14030411/s1, Figure S1: Bland–Altman plots illustrating intraobserver reproducibility, interobserver reproducibility, and intermethod agreement for myocardial transverse strain (TrS) of the left ventricle (in ToF and controls) or the systemic ventricle (in Fontan patients); Figure S2: Bland–Altman plots illustrating intraobserver reproducibility, interobserver reproducibility, and intermethod agreement for endocardial transverse strain (TrS) of the right ventricle (in ToF and controls) or the hypoplastic ventricle (in Fontan patients); Table S1: Anatomical and clinical characteristics of the Fontan cohort, presented as number (percentage) or mean ± standard deviation. Table S2: Intraobserver reproducibility of myocardial TrS, reported as mean ± SD, in the left ventricle in ToF and controls and in the systemic ventricle in Fontan patients; Table S3: Interobserver reproducibility of myocardial TrS, reported as mean ± SD, in the left ventricle in ToF and controls and in the systemic ventricle in Fontan patients; Table S4: Intermethod agreement between myocardial GLS and TrS for the left ventricle in ToF and controls and in the systemic ventricle in Fontan patients.

## Abbreviations

The following abbreviations are used in this manuscript:

BSA	Body Surface Area
CHD	Congenital Heart Disease
CMR	Cardiovascular Magnetic Resonance
ESV	End-Systolic Volume
EF	Ejection Fraction
EDV	End-Diastolic Volume
GLS	Global Longitudinal Strain
HV	Hypoplastic Ventricle
LAX	Long-Axis
LV	Left Ventricle
RV	Right Ventricle
SAX	Short-Axis
SD	Standard Deviation
SV	Systemic Ventricle
ToF	Tetralogy of Fallot
TrS	Transverse strain
