# Supplementary material for: Strain Analysis from Transverse CMR Cine Imaging in Congenital Heart Disease: Feasibility, Reproducibility, and Comparison to Global Longitudinal Strain
Source: Healthcare (Basel). 2026 Feb 6;14(3):411. doi: 10.3390/healthcare14030411 (PMC12897395; doi:10.3390/healthcare14030411)
Supplement: Supplementary file 1 [file healthcare-14-00411-s001.zip › healthcare-4079084-supplementary.pdf]

Supplementary Materials

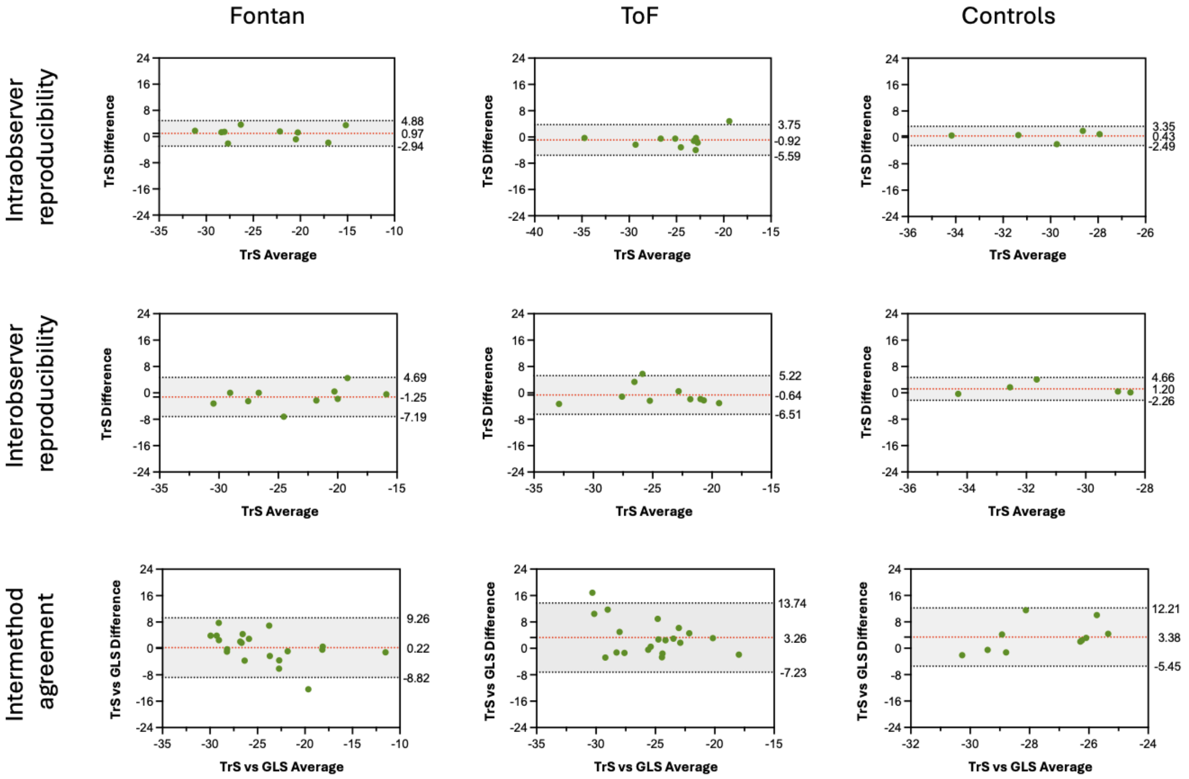

Figure S1. Bland–Altman plots illustrating intraobserver reproducibility, interobserver reproducibility, and intermethod agreement for myocardial transverse strain (TrS) of the left ventricle (in ToF and controls) or the systemic ventricle (in Fontan patients). Each plot displays the mean difference (red dashed line) and 95% limits of agreement (black dotted lines).

GLS = global longitudinal strain; ToF = tetralogy of Fallot; TrS = transverse-strain.

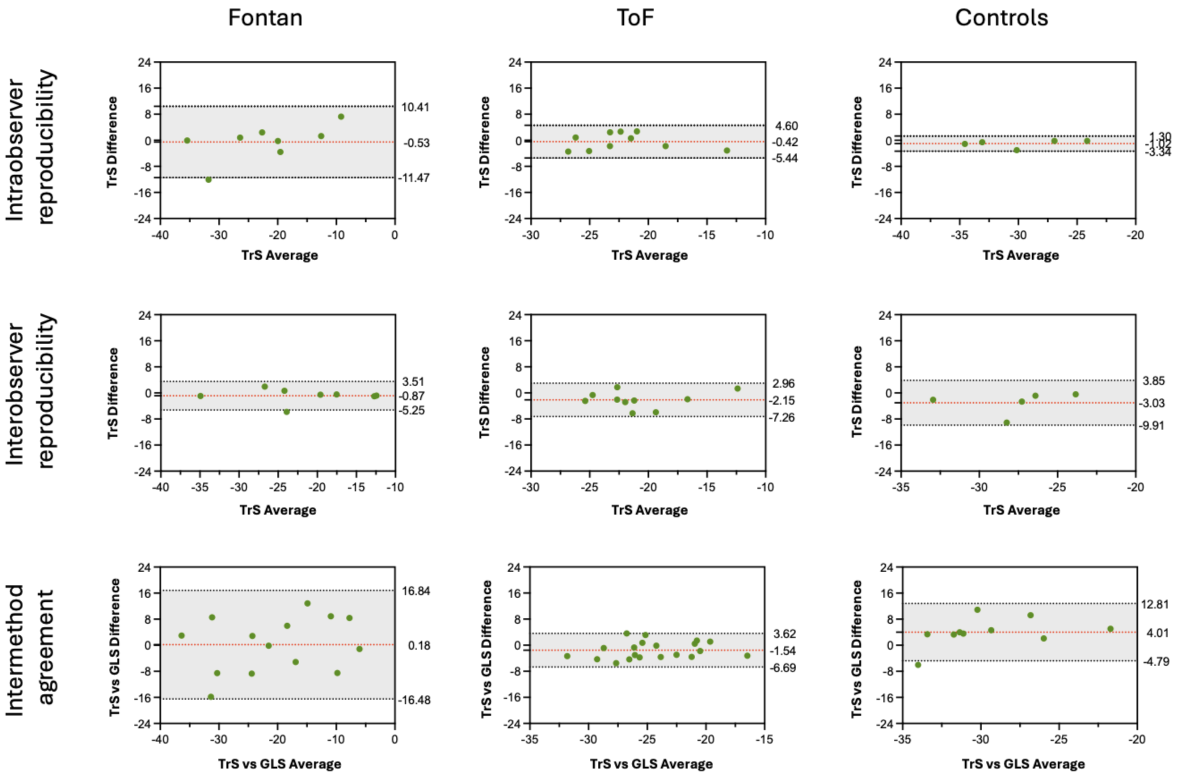

Figure S2. Bland–Altman plots illustrating intraobserver reproducibility, interobserver reproducibility, and intermethod agreement for endocardial transverse strain (TrS) of the right ventricle (in ToF and controls) or the hypoplastic ventricle (in Fontan patients). Each plot displays the mean difference (red dashed line) and 95% limits of agreement (black dotted lines).

GLS = global longitudinal strain; ToF = tetralogy of Fallot; TrS = transverse strain

Table S1. Anatomical and clinical characteristics of the Fontan cohort (n = 20).

Fontan cohort characteristics	n (%)
Fontan modification	
Extracardiac conduit	17 (85%)
Intracardiac Fontan	3 (15%)
Systemic ventricle morphology	
Systemic left ventricle	15 (75%)
Systemic right ventricle	5 (25%)
Primary fenestration	
Yes	8 (40%)
No	12 (60%)
Heterotaxy	
Yes	2 (10%)
No	18 (90%)
Underlying diagnosis	
Tricuspid atresia	8 (40%)
Pulmonary atresia	4 (20%)
Double outlet right ventricle	3 (15%)
Unbalanced atrioventricular septal defect	2 (10%)
Double inlet left ventricle	1 (5%)
Hypoplastic left heart syndrome	1 (5%)
Other (hypoplastic right heart syndrome)	1 (5%)
Fontan-related complications	
Protein-losing enteropathy	1 (5%)
Arrhythmia	2 (10%)
Liver cirrhosis	2 (10%)
Advanced heart failure (NYHA III/IV)	2 (10%)
Fontan failure syndrome	2 (10%)
Cardiopulmonary exercise testing (n=10)	
VO_2max (ml/kg/min), mean ± SD	23.8 ± 4.6

Data are presented as number (percentage) or mean ± standard deviation, as appropriate. Functional parameters are reported separately in Table 1. Fontan-related complications were extracted from clinical records; categories are not mutually exclusive.

Table S2. Intraobserver reproducibility of **myocardial TrS,** reported as mean ± SD, in the left ventricle in ToF and controls and in the systemic ventricle in Fontan patients.

Group	1st meas. TrS	2nd meas. TrS	Mean Difference	Limits of Agreement	ICC (95%CI)	r	p-value
**Fontan**	-23.20 ± 5.42	-24.16 ± 5.54	0.97	-2.94 - 4.88	0.93 (0.76-0.98)	0.93	<0.001
**ToF**	-25.62 ± 4.72	-24.70 ± 4.13	-0.92	-5.59 - 3.75	0.86 (0.52 - 0.96)	0.86	0.001
**Controls**	-30.15 ± 2.68	-30.58 ± 2.51	0.43	-2.49 - 3.35	0.84 (0.07-0.98)	0.84	0.08

ICC = intraclass correlation coefficient; meas. = measurement; r = Pearson's correlation coefficient; SD = standard deviation; ToF = tetralogy of Fallot; TrS = transverse strain.

Table S3. Interobserver reproducibility of **myocardial TrS,** reported as mean ± SD, in the left ventricle in ToF and controls and in the systemic ventricle in Fontan patients.

Group	1st obs. TrS	2nd obs. TrS	Mean Difference	Limits of Agreement	ICC (95%CI)	r	p-value
**Fontan**	-24.16 ± 5.54	-22.91 ± 4.51	-1.25	-7.19 - 4.69	0.82 (0.43-0.95)	0.84	0.002
**ToF**	-24.70 ± 4.13	-24.06 ± 4.54	-0.64	-6.51 - 5.23	0.76 (0.30-0.94)	0.77	0.01
**Controls**	-30.58 ± 2.51	-31.78 ± 2.71	1.20	-2.26 - 4.66	0.77 (-0.11-0.97)	0.77	0.12

ICC = intraclass correlation coefficient; obs. = observer; r = Pearson's correlation coefficient; SD = standard deviation; ToF = tetralogy of Fallot; TrS = transverse strain.

Table S4. Intermethod agreement between **myocardial GLS and TrS** for the left ventricle in ToF and controls and in the systemic ventricle in Fontan patients.

Group	GLS (mean ± SD)	TrS (mean ± SD)	Mean Difference	Limits of Agreement	ICC (95% CI)	r	p-value
**Fontan**	-24.30 ± 4.24	-24.53 ± 6.15	0.22	-8.82 - 9.26	0.62 (0.25 - 0.83)	0.66	0.001
**ToF**	-23.67 ± 3.35	-26.92 ± 4.99	3.25	-7.23 - 13.74	0.21 (-0.25 - 0.59)	0.23	0.34
**Controls**	-25.83 ± 3.45	-29.21 ± 2.03	3.38	-5.45 - 12.21	-0.27 (-0.75 - 0.40)	-0.31	0.38

GLS = global longitudinal strain; ICC = intraclass correlation coefficient; r = Pearson's correlation coefficient; SD = standard deviation; ToF = tetralogy of Fallot; TrS = transverse strain.
